# Interference without interferon: interferon-independent induction of interferon-stimulated genes and its role in cellular innate immunity

**DOI:** 10.1128/mbio.02582-24

**Published:** 2024-09-20

**Authors:** Shachee Swaraj, Shashank Tripathi

**Affiliations:** 1 Emerging Viral Pathogens Laboratory, Centre for Infectious Disease Research, Indian Institute of Science, Bengaluru, India; 2 Microbiology & Cell Biology Department, Biological Sciences Division, Indian Institute of Science, Bengaluru, India; Albert Einstein College of Medicine, Bronx, New York, USA

**Keywords:** viral immunity, innate immunity, interferons, ISG induction, interferon-independent immunity, STAT signaling, JAK kinases

## Abstract

Interferons (IFNs) are multifaceted proteins that play pivotal roles in orchestrating robust antiviral immune responses and modulating the intricate landscape of host immunity. The major signaling pathway activated by IFNs is the JAK/STAT (Janus kinase/signal transducer and activator of transcription) pathway, which leads to the transcription of a battery of genes, collectively known as IFN-stimulated genes (ISGs). While the well-established role of IFNs in coordinating the innate immune response against viral infections is widely acknowledged, recent years have provided a more distinct comprehension of the functional significance attributed to non-canonical, IFN-independent induction of ISGs. In this review, we summarize the non-conventional signaling pathways of ISG induction. These alternative pathways offer new avenues for developing antiviral strategies or immunomodulation in various diseases.

## INTRODUCTION

Cellular innate immunity poses the first hurdle in the quest for viral pathogens to establish infection. It starts with the detection of pathogen-associated molecular patterns (PAMPs) by cellular pathogen recognition receptors (PRRs) leading to interferon (IFN) induction (1, 2). Subsequent IFN signaling then produces IFN-stimulated genes (ISGs) that block virus replication and trigger adaptive immune responses (1, 2). Antiviral action of ISGs is usually broad spectrum but with no memory (3). PRRs, upon detecting viral PAMPs like their genome (RNA or DNA) or structural components, activate transcription factors (TFs) such as IFN regulatory factors (IRFs) IRF3 and IRF7, nuclear factor-κB (NF-κB), and activating transcription factor-2 (ATF-2)/c-Jun (1, 2, 4). These TFs translocate into the nucleus to stimulate IFNs and proinflammatory cytokines that are subsequently released by the virus-infected cells into their microenvironment (Fig. 2) (2, 5, 6). This establishes an antiviral state in the cells neighboring the site of infection (6).

IFNs are crucial in bridging innate and adaptive immunity and are classified into three types based on their signaling receptors (1, 7). Type-I IFNs (IFN-α, IFN-β, IFN-ε, IFN-κ, and IFN-ω) function via heterodimeric cell surface receptors IFNAR1/2 in a paracrine or autocrine manner (7–10). Type-II IFN (IFN-γ) binds to IFNGR1/2 (11, 12), while type-III IFNs (IFN-λ1/2/3) utilize IL10R2 and IFNLR1 for downstream signaling (13–15). All IFNs activate the JAK/STAT pathway (16), with type-II IFNs relying on STAT1 homodimers, known as the γ-activated factor (GAF) (17), for this purpose, while type-I and type-III IFN signaling engage phosphorylated STAT1 (pSTAT1) and pSTAT2 heterodimer followed by its interaction with IRF9 to form a heterotrimeric complex called ISG factor 3 (ISGF3) (10, 18–23). It is responsible for the transcription of ISGs by binding to the ISRE (IFN-stimulated response element) sequence (consensus sequence: ^A^/_G_NGAAANNGAAACT) and recruiting RNA polymerase (20–22, 24–28). Signals from all the IFNs converge to induce ISGs that assume various roles (Fig. 2) (29, 30). Some act as direct antiviral effectors that target various stages of the pathogen lifecycle, like Mx, CH25H, IFITM proteins, OAS and RNaseL, TRIM proteins, viperin, tetherin, and so on (29, 30), while others like retinoic acid-inducible gene 1 (RIG-I)-like receptors (RLRs), AIM2-like receptors (ALRs), toll-like receptors (TLRs), IRF1/7/9, STAT1, STAT2, and cyclic GMP-AMP (cGAMP) synthase (cGAS) can enhance the innate pathogen-detection pathways (30). Conversely, a few ISGs, such as USP18 and SOCS protein, exert negative regulatory effects on innate immune signaling to maintain cellular homeostasis (Fig. 2) (30).

Conventionally, ISGs are activated by the IFN-induced JAK/STAT pathway during viral infections (16). Yet, increasing evidences underscore a significantly more intricate process of activation and function that extends beyond this traditional paradigm. In recent decades, mounting research has shown that a subset of ISGs can be directly induced without IFN signaling (31–40). In contrast to canonical ISG induction, which involves a series of temporal events, non-canonical ISG induction triggers an early and rapid antiviral response, bypassing the IFN-mediated pathway, and plays a crucial role in shaping innate immunity and inflammation (Fig. 2) (33, 41–43). In this comprehensive review, we have summarized the studies highlighting non-canonical IFN-independent ISG induction and its contribution to cell-intrinsic immunity.

## THE NON-CANONICAL IFN-INDEPENDENT ISG INDUCTION

**Fig 1:**
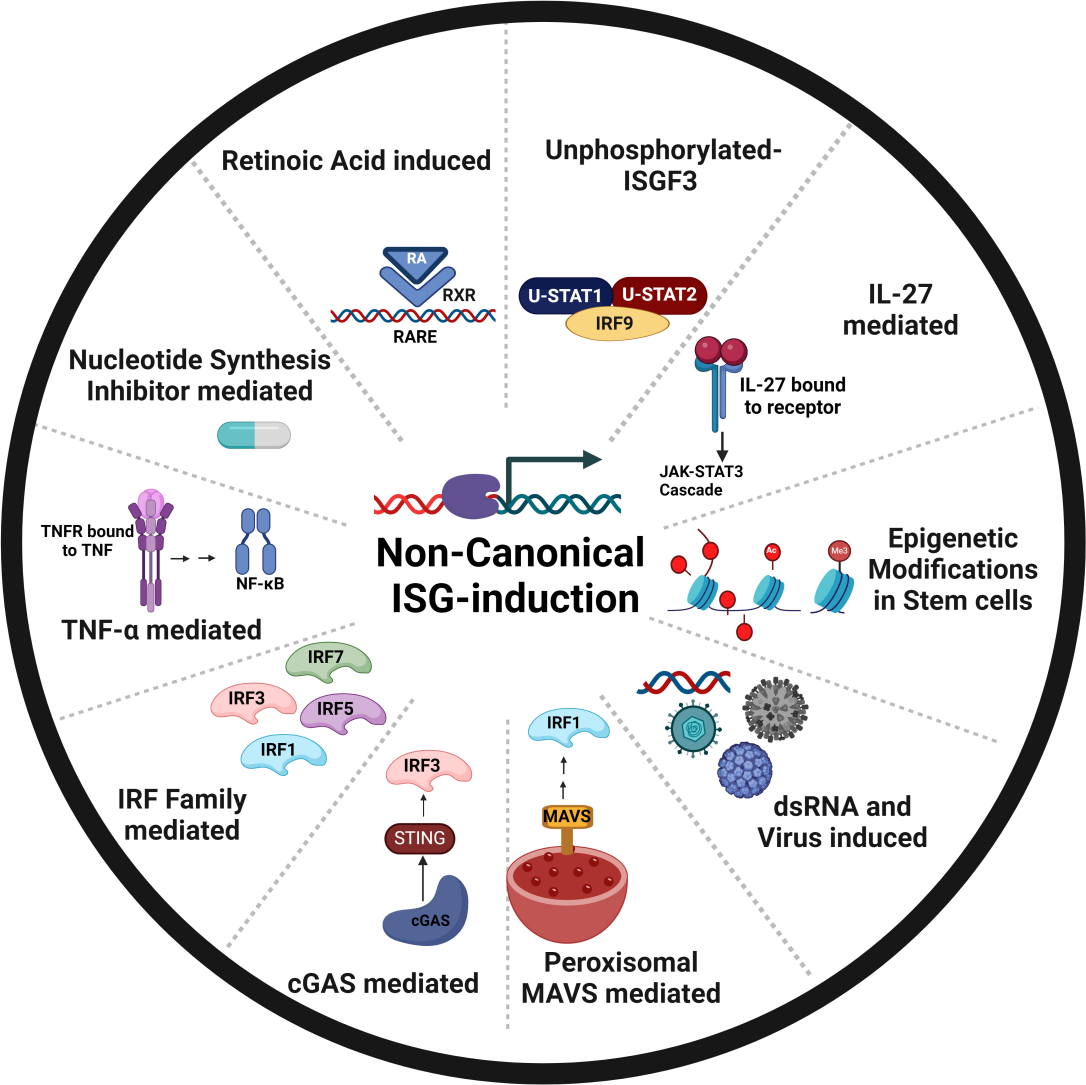
Schematic representation of various non-canonical ISG induction mechanisms. This figure outlines the alternative stimuli that can trigger ISG transcription, bypassing the canonical IFN-JAK-STAT axis.

**Fig 2:**
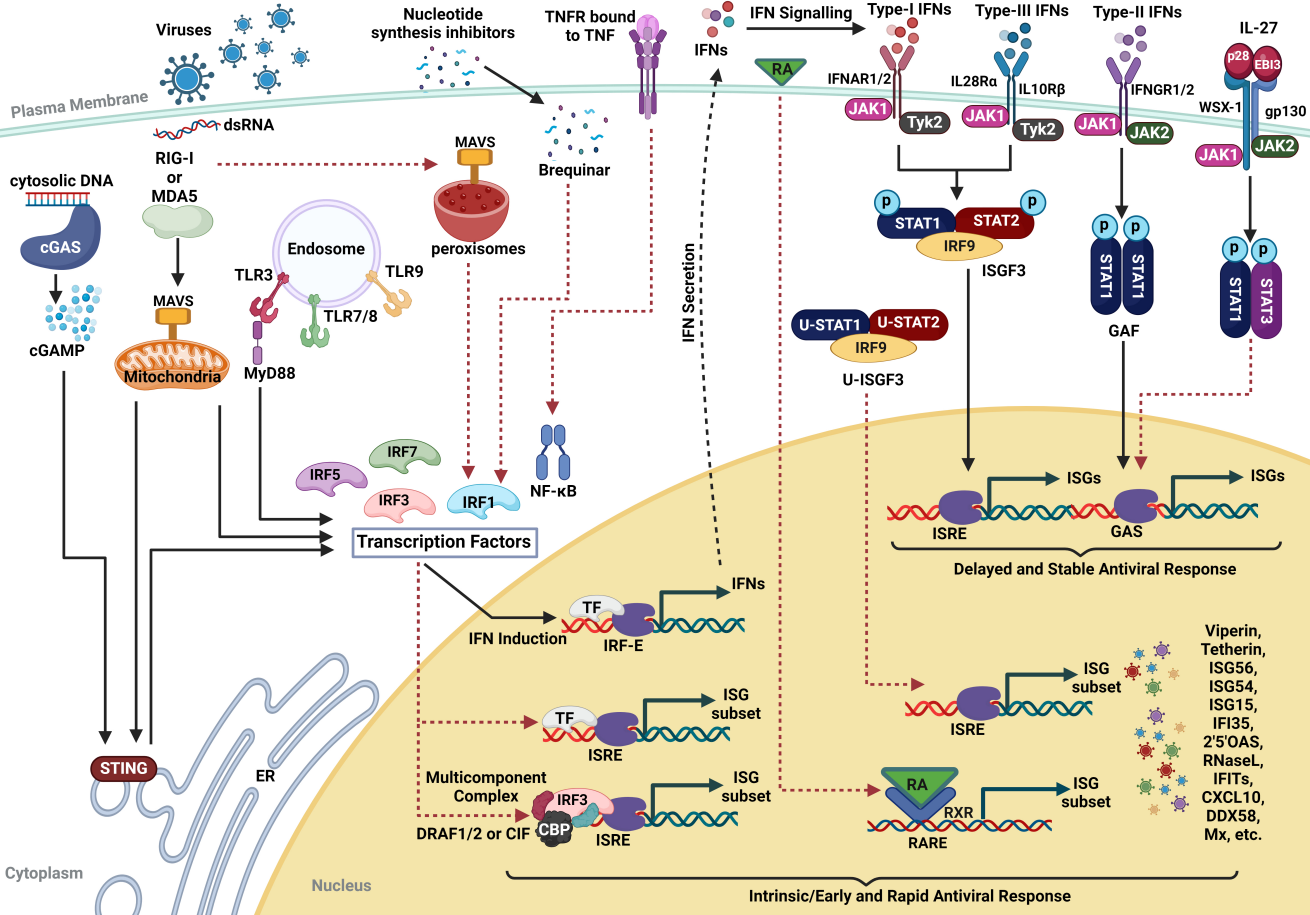
Canonical and non-canonical ISG induction pathways. Viruses are recognized by PRRs like RIG-I/MDA5, cGAS, or TLRs that activate TFs, including IRFs, NF-κB, and so on, via adaptor proteins including MAVS, MyD88, or STING. These TFs trigger IFNs and proinflammatory cytokines, leading to ISG induction via the JAK/STAT cascade. Unconventionally, these TFs can drive transcription of a subset of ISGs by directly binding to their ISREs upon immune stimulation. IL-27, pex-MAVS, U-ISGF3, RA, TNF-α, and NSI, like Brequinar, also drive ISG transcription via unique pathways. Solid black arrows: canonical signaling; dashed red arrows: non-canonical signaling. ER: endoplasmic reticulum.

Non-canonical induction of ISGs refers to pathways that operate independently of the IFN-JAK-STAT signaling. They can be triggered by various stimuli, such as cellular stress or viral infections (Fig. 2). In the following section, we provide a detailed account of various scenarios where ISGs are induced independent of IFN induction or signaling (Fig. 1).

### IFN-independent unphosphorylated ISGF3 (U-ISGF3)-mediated basal ISG induction

Phosphorylation of STATs is crucial for canonical activation of the JAK/STAT pathway by IFNs (16). However, studies suggest that STATs can also function without phosphorylation. U-STAT1 is capable of inducing ISRE promoter activity independent of pSTAT1 (44). In senescent cells, U-ISGF3 is the primary activator of IFN-independent constitutive ISG expression (45). Wang et al. reported that U-ISGF3, composed of U-STAT1, U-STAT2, and IRF9, is continuously shuttled between the nucleus and cytoplasm across cell types, activating ISREs in an IFN-independent manner (46). The baseline expression of ISGs like IFIT2, PKR, IFIH1, DDX58, ISG15, IRF1, IFITM1/3, IRF9, and STAT1/2 was robustly detected across multiple cell lines and organoid models without any immune stimuli (46). Cheon et al. demonstrated that the IFN-dependent canonical ISGF3 triggers early ISG transcription, including STAT1, STAT2, and IRF9. However, later, when the pSTAT1 levels drop down, the elevated levels of IRF9, along with U-STAT1 and U-STAT2, promote a second prolonged phase of ISG expression via U-ISGF3 (47, 48). Although the functions of U-ISGF3 are not fully understood, it is speculated to play a role in sustaining basal ISG induction to promote resistance to viruses (47–49). Sung et al. also noted that, during chronic hepatitis C virus (HCV) infection, U-ISGF3, and not pSTAT1, was significantly elevated in response to IFN-λ and IFN-β, prolonging ISG expression and promoting viral resistance (50). Additionally, a non-canonical STAT2-IRF9 complex also drives STAT1-independent ISG expression, contributing to antiviral immunity (51–55). Other than an early moderate activation by ISGF3 and a strong activation later by U-ISGF3, the interleukin-6 levels were also elevated by the U-STAT2-IRF9 complex in cooperation with NF-κB (56). However, U-ISGF3-mediated ISG induction is possibly cell-type specific since there's no evidence of it in hematopoietic cell lines, like macrophages, where the ISG profile is primarily shaped by IFNs and IRF1 (57). These insights underscore the critical functions of STAT signaling beyond the canonical IFN- and phosphorylation-dependent pathways.

### Interleukin-27 (IL-27)-mediated ISG induction

IL-27, produced upon TLR activation, is a cytokine composed of two subunits, IL-27p28 and Epstein-Barr virus-induced gene 3 (EBI3) (58, 59). It acts via a heterodimeric receptor involving WSX-1 and glycoprotein-130, leading to the activation of JAK1/2 that, in turn, phosphorylates STAT1/3 (59, 60) (Fig. 2). These TFs translocate into the nucleus to induce transcription of several genes, including IFNs, antiviral ISGs, immunomodulatory cytokines and chemokines, and a few microRNAs (61). IL-27 elicits an antiviral response by stimulating ISG expression in both IFN-dependent and IFN-independent manner (61). This IFN-independent activity of IL-27 has been demonstrated against Chikungunya virus (CHIKV) (62), Zika virus (ZIKV) (63, 64), herpes simplex virus (HSV) (65), HCV (66), human immunodeficiency virus (HIV) (67), Influenza virus (68), coxsackievirus B3 (CVB3) (69), and severe acute respiratory syndrome coronavirus 2 (SARS-CoV-2) (70). For instance, in ZIKV-infected mice, the epidermal keratinocytes induce the expression of OAS1, OAS2, OAS3, OASL, and MX1 in an IL27RA/STAT1-dependent but IFNAR1/STAT2-independent manner upon IL-27 treatment, which is crucial for mice survival (63). IL-27 has been reported to induce ISGs like PKR, IFITMs, MX1/2, and RSAD2 in macrophages during CHIKV infection (62), BST2 in human monocytes and CD4 T cells during HIV infection (71), and GBP1, IFIT2, IFITM1, ISG15, MX1, OAS2, and viperin in human macrophages stimulated with the SARS-CoV-2 spike protein in the absence of IFNs (70). Beyond immune cells, IL-27 triggers the expression of IRF1/9, MxA, GBP2, and STAT1/2 in liver cells treated with anti-type-I IFN antibodies (68). IL-27 treatment activates STAT1 as early as 10 minutes post-treatment in hepatic stellate cells (72) and STAT1/3 within 20 minutes in HeLa and glioblastoma cells before any protein synthesis kicks in, further validating IFN-independent activity, via pSTAT1 and pSTAT3 (65). These findings suggest IL-27 as a versatile cytokine with potent antiviral properties, working through both IFN-dependent and IFN-independent mechanisms, and as a promising therapeutic agent against various viral infections.

### Intrinsic IFN-independent ISG expression in stem cells

Embryonic stem cells (ESCs) and induced pluripotent stem cells (iPSCs) are not responsive to IFN stimulation (73), but they inherently express certain antiviral ISGs (74, 75). This ISG expression varies by stem cell type, decreases with differentiation, and is conserved across mammals (74). ISGs like IFITMs, OCT4, EIF3L, BST2, and many others are highly expressed in all stem cells, even in the presence of IFN-neutralizing antibodies (74). IFITM triple knockout ESCs (IFITM1/2/3^−^/^−^) show increased viral susceptibility to influenza A virus (IAV), respiratory syncytial virus (RSV), human parainfluenza virus 3 (hPIV3), yellow fever virus (YFV), dengue virus (DENV), West Nile virus (WNV), ZIKV, and Newcastle disease virus (NDV) (74). During stem cell differentiation, IRF1 is transiently induced and binds to promoters of certain ISGs such as DDX58, DDX60, TRIM56, and viperin, promoting their expression (76). In somatic cells, IRF1 supports IFN-independent ISG induction via epigenetic mechanisms (77). In stem cells that lack IFN responsiveness, similar histone markings like H3K4me3 and H3K27ac regulate ISG expression, which decreases upon differentiation (74), possibly through IRF1. These epigenetic modifications are abundant in the promoters of highly expressed ISGs (e.g., IFITM3 and IGFBP2), compared to unexpressed ones (e.g., MX1 and OAS1) in ESCs (74). The shared transcriptional regulation of IFN-independent ISGs between stem cells and differentiated cells offers insights into the evolution of antiviral defense during differentiation.

### Double-stranded RNA (dsRNA)-mediated induction of ISGs

Recent studies have shown that the viral dsRNA can induce ISGs in IFN-deficient systems. A full repertoire of dsRNA-stimulated genes (DSGs) was identified by cDNA microarray hybridization screening in GRE cells deficient in IFN genes. The screen detected 175 DSGs, a subset of which belonged to varied immune pathways (78). IFN-independent ISG activation occurs through the involvement of TFs such as IRFs, NF-κB, and associated coactivators (31, 37, 38, 43, 79–87). For instance, dsRNA-induced RIG-I triggers two distinct sets of ISGs by independent pathways, both of which contribute co-ordinately to the anti-hepatitis E virus (HEV) activity. One set, including, STAT1, IRF1, and IRF9, depends on STAT1 phosphorylation while the other set, including IFIT1, IFIH1, and RANTES, does not (40). Another study on RLR signaling-mediated anti-CVB3 response suggested that melanoma differentiation-associated protein 5 (MDA5), but not RIG-I, could mount an anti-CVB3 response independent of type-I and type-III IFNs, suggesting a virus- and cell-type-specific responses by RLRs (88). ZAP, an antiviral effector ISG that inhibits replication of filoviruses (89), retroviruses (90), and alphaviruses (91–93), is induced by viral infection and dsRNA via TLR3, RIG-I/MDA5, and IRF3 axis in type-I and type-III IFN signaling-deficient cells (32). Synthetic dsRNA analog, polyinosinic acid–polycytidylic acid (poly I:C), induces ISG15 and ISG43 in porcine kidney cells along with a few PRRs, including TLR3, DDX58, and IFIH1 without inducing IFN (94). ISG56 can be induced independently of IFN, during viral infection in cells lacking Jak1 (U4C and P2.1 cells), requiring IRF3 nuclear translocation (80). Furthermore, dsRNA-dependent induction of ISG56 bypasses IFN production and requires IRF1 and functional STAT1-α (95). However, a mutation in its ISRE sequence abolishes both IFN-α and dsRNA-mediated ISG induction, emphasizing the critical role of the cis-acting ISRE elements (95). IFN-independent immune activation in mouse fibroblasts is influenced by dsRNA length, with short dsRNA triggering ISGs through mitochondrial antiviral signaling protein (MAVS) and IRF3, while long dsRNA operating independently of these factors (34). This indicates a possible new pathway for ISG regulation by dsRNA.

### Virus infection-induced ISGs or virus-stimulated genes (VSGs)

Viruses have evolved a multitude of strategies to evade canonical IFN response (96). However, they remain sensitive to immune activation even in IFN-deficient systems, suggesting the existence of IFN-independent antiviral pathways. For example, in SARS-CoV-2-infected cells despite the inhibition of the RLR pathway, IFN production, and STAT1 phosphorylation, ISGs like OAS2 and a few IFITs are induced (97). Such genes, often called VSGs, are triggered upon viral infection that partially overlaps with the IFN-induced transcriptome (98). For instance, measles virus (MeV) infection moderately induces OAS1, MxA, STAT1, STAT2, IRF9, RIG-I, MDA5, IRF3, CXCL10, and IL6 genes without inducing any detectable type-I and type-III IFNs, limiting virus spread (99). VSGs are also reported to be induced early after the infection but before IFN induction (98), likely via IFN-independent pathways employing TFs like IRFs or NF-κB. In human rhinovirus (HRV-16)-infected human bronchial epithelial cells, CXCL10 and ISG15 can be induced independently of IFNs, through direct induction of IRF1 (100, 101). This is supported by previous reports demonstrating robust upregulation of IRF1 and IRF7 during HRV infection (102, 103). Kim et al. studied transcription activation in response to IAV and IBV, independent of IFN-α/β as part of an early antiviral response, which was IRF3-dependent (79). During acute IAV lung infection in mice, RIG-I and MDA5 are induced independently of IFN signaling, possibly regulated by Duox, the major nicotinamide adenine dinucleotide phosphate oxidase (Nox) isoform-derived reactive oxygen species (ROS) (104). Studies with a variety of enveloped viruses have shown that several ISGs can be induced to establish an early antiviral response (41, 42, 105–108), many of which are transcribed by IRFs. Noyce et al. described a novel PI3 kinase pivotal for antiviral state induction (109). During human cytomegalovirus (HCMV) infection, ISGs like viperin, ISG15, IFIT1/2/3, and Mx1/2 are upregulated in an IRF3-dependent but a STAT1-independent manner (110), potentially through its immediate early gene 1 (IE1) protein (111) or a distinct ISRE-recognizing complex involving histone acetyltransferase CBP and IRF3, which is triggered by viral entry without requiring genome replication (112). This is supported by evidence that ISG induction can occur without viral replication, as viral proteins alone can trigger this process (31, 41–43, 86, 107, 113–117). Similar multicomponent complexes, like cytomegalovirus-induced ISRE-binding factor (CIF) and dsRNA-activated factors 1 and 2 (DRAF1 and DRAF2) (Fig. 2), are involved in activating ISGs by binding directly to ISREs (36, 81, 118, 119). Additionally, DRAF1 promotes apoptosis in response to viral infection without IFN signaling (120).

Flaviviruses can evade IFN-mediated antiviral signaling (121, 122), yet a cohort of ISGs is triggered during DENV (123), WNV (33, 124), and lymphocytic choriomeningitis virus (LCMV) infections (124). ISGs, like OAS1a/b, IRF7, and IRF1, were upregulated in WNV-infected STAT1^−^/^−^, STAT2^−^/^−^, and IFNAR^−^/^−^ MEFs (33). Functional assays revealed the involvement of an alternate TF complex containing NF-κB along with IRF3, IRF5, or IRF7 and possibly additional unknown factors that regulate ISG transcription via two ISRE motifs in the ISG promoters (125). These findings highlight diverse mechanisms involving TFs like IRF3, IRF5, IRF7, and IRF1, as well as other factors such as PI3 kinase and DRAFs, contributing to the induction of ISGs and early antiviral responses against different viruses, in an IFN-independent manner.

### Peroxisomal MAVS (pex-MAVS)-mediated ISG induction

MAVS is a key innate immune adaptor anchored to the mitochondrial membrane, classically relaying RLR signaling to TFs for type-I IFN induction (Fig. 2) (126). Work by Dixit et.al. revealed that MAVS also resides on peroxisomes, which rapidly induces a transient ISG expression without IFN induction (127–130). Contrary to pex-MAVS, mitochondrial MAVS promotes a stable and long-lasting induction of both IFN and ISG expression, albeit with delayed kinetics. Also, in their analysis of MAVS downstream signaling, the authors found that TRAF3, TRAF6, and IRF3 were necessary for signaling from both mitochondria and peroxisomes; however, pex-MAVS specifically activated IRF1 (127). This supports the other findings that describe IRF1's IFN-independent ability to induce an antiviral program (39). Zheng et al. described the early induction of viperin upon HSV-1 infection in HEK293 cells independent of type-I and type-III IFNs via pex-MAVS signaling (129). Other groups have also recognized peroxisomes as key organelles for type-I and type-III IFN-dependent and IFN-independent antiviral signaling (128, 130). Interestingly, several publications report pex-MAVS as targets for viral antagonism. For instance, viruses cleave pex-MAVS or disrupt peroxisomal biogenesis impeding peroxisomal MAVS-mediated antiviral pathways (129–134). These findings confirm peroxisomes as a pleiotropic platform for antiviral signaling. However, more insight into the peroxisomal signaling pathway and the mechanisms underlying IRF1 activation are required.

### cGAS-mediated ISG induction

cGAS is a cytosolic DNA sensor that catalyzes the production of a secondary messenger, cGAMP, which then triggers the stimulator of IFN genes (STING)-IRF3 signaling axis for type-I IFN production and antiviral signaling (Fig. 2) (135, 136). However, research indicates that STING can mediate antiviral responses in an IRF3/IFN-independent manner, likely by inducing autophagy and/or apoptosis across different species (137–145). STING mutant mice, incapable of IFN induction, promote anti-HSV1 and antitumor responses, highlighting the role of autophagy or a similar process in these defenses (145), possibly by direct interaction with microtubule-associated protein 1L chain 3 (LC3) and in a non-canonical, ATG5-dependent pathway (143, 144, 146). Schoggins et al. noted that cGAS induced an antiviral response against multiple RNA viruses in STAT1^−^/^−^ fibroblasts, despite it being a DNA sensor (147). Lentiviral transduction-driven overexpression of cGAS in STAT1^−^/^−^ fibroblasts activated a STING-IRF3-mediated antiviral program independent of IFN signaling as determined by microarray analysis (147). STING also strengthens anti-hantaviral immunity by inducing autophagy via RIG-I, operating independently of canonical IFN responses (148). Overall, cGAS and STING play a crucial role in antiviral immunity against both DNA and RNA viruses through multiple pathways beyond traditional type-I IFN production.

### IRFs as direct inducers of ISGs

IRFs are a family of TFs involved in regulating the innate IFN response and other cellular processes like inflammation, tumorigenesis, and cancer (149–151). IRFs typically share a unique helix-turn-helix DNA-binding motif that recognizes the IRF response element (IRF-E) in the upstream regulatory elements of IFN genes (152, 153). While some IRFs activate transcription (e.g., IRF1, IRF3, and IRF9), others suppress it (e.g., IRF8), with variations in DNA binding patterns within the consensus sequence: 5′ AANNGAAANNGAAA3′ (154–158). On the other hand, IRF2, IRF4, IRF5, and IRF7 are capable of both activating and repressing transcription (154–159). Some IRFs directly induce transcription of a subset of ISGs, owing to the sequence similarity between IRF-E and ISRE (160). This allows IRFs to bind directly to ISREs upstream of certain ISGs, providing functional redundancy (81, 87, 160, 161). Despite this, not all ISGF3-induced genes are triggered by all IRFs. This specificity arises from the unique sequence requirements of each of the IRFs and ISGF3 both within the core sequence and the flanking regions in the ISG promoters (160). Consequently, this allows the induction of unique transcriptional profiles by each of the IRFs only partly overlapping with the ISGF3 transcriptome (160).

#### IRF1 mediated

IRF1 is a key protein in the IRF family that was the first to be identified as binding to the IFN-β promoter (162, 163). It is an ISG, differentially upregulated by IFNs, dsRNA, or viruses across various cell types (161, 164–166). In respiratory epithelial cells, it is induced early by IFN-β but minimally by IFN-λ, while in hepatocyte cell lines, it remains uninduced by IFN-λ, owing to its cell-type-specific receptor abundance (57, 167). After IRF1's IFN-independent antiviral functions came to light (161, 168), several reports showed its overexpression to protect against various viruses (77, 169–173). It has been reported to bind to the PRD-I regulatory element of the IFN-β gene and the central region of the 15 base pairs (bp) ISRE sequence upstream of ISGs required for their transcription (161). IRF1, but not IFN, was proved indispensable for the expression of viperin, during vesicular stomatitis virus (VSV) infection in embryonic fibroblasts (39) and HSV infection in mouse fibroblasts (174). Additionally, IRF1 activated other ISGs such as iNOS (175) and CH25H (173), by directly binding to the two proximal IRF-Es in the ISG promoters, instigating their IFN-independent enhancement (39, 175). In another investigation, Xu et al. revealed that IRF1 suppresses HEV replication in Huh7 cells via a STAT1-dependent, IFN-independent mechanism (176). Additionally, IRF1 was shown to epigenetically control the transcription of certain ISGs, protecting against VSV infection in human bronchial epithelial cells (77). Mechanistically, IRF1 modulates the H3K4me1 modification at ISG promoter regions, especially PRRs, thus regulating their constitutive early expression (77). H3K4me1 helps to bring specific chromatin readers, such as bromodomain-containing protein 4 (BRD4), to identify chromatin modification and histone tails (177), leading to ISG transcription.

Microarray analysis by Schoggins et al. to transcriptionally profile the genes induced in IRF1-transduced Huh-7 cells and STAT1^−^/^−^ fibroblasts revealed induction of several ISGs that inhibited HCV, HIV-I, YFV, WNV, CHIKV, and Venezuelan equine encephalitis virus (VEEV) without inducing any IFN genes (171). Under physiological conditions, constitutively expressed IRF1 localizes in the nucleus and triggers basal expression of several ISGs that ensure an early and immediate restriction of multiple positive-sense RNA viruses, including hepatitis A virus (HAV) and HCV, DENV, and ZIKV, in hepatocytes independently of IFN signaling (172). IRF1 also inhibits duck Tembusu virus (DTMUV) in chicken fibroblasts and duck embryo fibroblasts by direct ISG induction in the absence of IRF7 and type-I IFNs (178). While, in muscle cells, IRF1 inhibits CHIKV and Ross River virus (RRV) in their initial stages of infection (170), it targets VSV in later stages, preventing fatal neurotropic infection in the central nervous system (CNS) via IFN-independent antiviral program (169). Furthermore, IRF1 coordinates with IRF8, forming a regulome to regulate gene transcription in primary macrophages under basal conditions and following IFN-γ stimulation (179). Overall IRF1 mediates unique and layered antiviral responses against multiple viruses that bypass the canonical IFN program of ISG induction through multiple mechanisms, including STAT-dependent or STAT-independent pathways, likely influenced by cell type and specific conditions.

#### IRF3 mediated

IRF3 is a constitutively expressed TF that stays in the cytoplasm in a latent state but is promptly activated by phosphorylation upon virus detection (180, 181). Activated IRF3 dimerizes, translocates to the nucleus, and associates with CBP/p300 to induce type-I IFN (180–182). Additionally, IRF3 forms an enhanceosome complex with NF-κB and ATF-2/c-Jun that triggers the IFN-β production (183–185). Besides inducing IFNs, IRF3 directly binds to ISREs, triggering ISG induction (81, 87). DNA microarray analysis identified ISGs, including ISG54, ISG56, ISG60, GBP, 2′5′OAS, IFI44, and ISG15, among the 277 differentially expressed genes (DEGs), that were IRF3 responsive in Jurkat T cells via direct binding of IRF3 to ISRE (186). Taniguchi's team demonstrated IFN-independent IRF3's role in regulating ISG54, GBP1, and ISG15 during NDV infection in IRF3-deficient MEFs (187). Microarray analysis on virus-infected wild type (WT) or IRF3^−^/^−^ MEFs detected a few IRF3-dependent genes, including IFP35, ISG20, I-8U, cig5/viperin, and ISG54, otherwise typically transcribed upon ISGF3 activation via IFN signaling (38). Although the underlying molecular mechanism responsible for such a distinct regulation of ISG expression by IRF3 is not fully elucidated, a few studies revealed that IRF3 has a well-defined ISRE DNA-binding site [GAAA(C/G)(C/G)GAAAN(T/C)] enabling it to activate a subset of ISGs (188). Additionally, the surrounding nucleotide sequences in the ISRE region may play a crucial role in IRF3-ISRE binding (118, 187). This knowledge confirms an implicit bypass to an early antiviral defense.

#### IRF5 mediated

IRF5 is a TF that regulates innate antiviral immunity as well as cancer, inflammation, cellular growth, and apoptosis (189–192). Despite being a robust inducer of IFN-β, it was not shown to trigger ISGs directly (193). Nonetheless, it possesses two nuclear localization signals and is detectable in the nucleus at basal levels, increasing upon virus infections (190, 194), hinting at its potential to induce genes independently of IFNs. Indeed, Nandakumar et al. unveiled an IFN-independent anti-HCV function of IRF5 in MEFs (35). Additionally, IRF5 overexpression in U3A cells lacking functional STAT1 stimulated numerous genes spanning various biological functions, including antiviral responses (195).

#### IRF7 mediated

Unlike IRF3, which is constitutively expressed, IRF7 is induced by IFNs in most of the cells except a few immune cells where its expression is constitutive (196). It is also activated by a virus-activated factor (VAF) complex consisting of IRF3, IRF7, and CBP/p300 independent of IFNs (197). Consequently, IRF3 governs the early IFN-independent antiviral signaling leading to robust IFN-β induction, while IRF7 levels, during this phase, remain low due to minimal IFN-α/β signaling. However, with subsequent IFN-α/β signaling, IRF7 levels increase in the later stage, collaborating with IRF3 to amplify IFN-α/β gene induction (198). Like IRF3, IRF7 recognizes IRF-E in target gene promoters but with greater promiscuity, suggesting broader DNA binding potency for stimulating IFNs and ISGs (188, 195). To impanel IRF7-induced IFN-independent ISGs, Schmid et al. performed transcriptional profiling of genes in the lung tissue obtained from IAV-infected B6.A2G-Mx1-IFNAR1^−^/^−^-IL28Rα^−^/^−^ mice (160). The screen revealed several virus-induced genes that were typical ISGs previously thought to be IFN signaling dependent. Molecular assays revealed that the IRF7 homodimer, rather than the IRF3-IRF7 heterodimer, predominantly induced most ISGs in an IFN-independent manner, with many genes being unique to the IRF7-induced transcriptome and a few overlapping with IRF3-dependent targets, highlighting a conserved functional redundancy among ISGF3, IRF3, and IRF7 (160).

### Tumor necrosis factor-α (TNF-α)-mediated ISG induction

TNF-α, initially recognized for its anti-tumor activities (199), has pleiotropic functions, including regulating inflammation, antiviral responses (200–202), and autoimmune diseases (203). Interactions between TNF-α and its receptor (TNFR) significantly influence the outcome of various viral infections by either aiding in virus control, contributing to immune-mediated pathology (204), or triggering a sustained expression of chemokines and STAT1-dependent ISGs (205). Previous investigations have noted interactions between TNF-α and the IFN signaling pathway to regulate the ISG transcription as a measure to restrict VSV (206), HCV (207), poxvirus (208), and respiratory virus infections (209). For decades, IFN-α has been a mainstay in treating chronic hepatitis B virus (HBV) and HCV and has been used off-label in certain cases of HEV infection (210–212). However, a recent study demonstrated that TNF-α treatment restricted HCV replication by 71 ± 2.4% and HEV replication by 41 ± 4.9% (213). Intriguingly, this effect was brought about by the induction of a panel of ISGs by TNF-α, independent of the canonical IFN and the JAK/STAT signaling cascade. This induction occurs via TNF-α-TNFR1 binding (213) activating the NF-κB protein complex. Bioinformatics analysis determined that the consensus DNA binding sequence of NF-κB (5′-GGGAA/CTTTCC-3′) (214) shows similarity to ISRE motifs upstream of ISGs (213). Molecular investigations uncovered that NF-κB directly binds to the ISRE region of the ISGs, triggering their transcription (213).

### Nucleotide synthesis inhibitors (NSI)-mediated ISG induction

*De novo* nucleotide biosynthesis is crucial for both host cell metabolism and viral replication, making enzymes in these pathways potential antiviral targets (215). Recent research has highlighted the interplay between nucleotide depletion and the cellular antiviral immune response involving the expression of ISGs. NSIs like ribavirin and mycophenolic acid (MPA) target IMP dehydrogenase (IMPDH) of the purine biosynthesis pathway, limiting viral replication through purine nucleotide depletion (216–220) and ISG induction (221, 222). Several NSIs demonstrate broad antiviral activity against YFV (223), CMV (224, 225), HBV (216), HCV (220, 222), BK virus (226), HEV (217, 221), and DENV (218, 219). Wang et al., using RT-PCR and ISRE-driven luciferase activity assay, demonstrated that, in HEV infection model, MPA and three other IMPDH inhibitors induced a panel of ISGs including IRF1/9/7, ISG15, IFI6, IFI27, CXCL10, MX1, DDX58, IFIT1/2, and STAT1 in an IFN-independent manner (221). Another NSI, tetrahydrobenzothiazole-based compound 1, displayed a robust broad-spectrum antiviral activity against ebolavirus, flaviviruses, alphaviruses, and influenza viruses by the induction of a variety of ISGs in IFN signaling-deficient systems in a cell-type-specific fashion (227). Brequinar and leflunomide, specific inhibitors of dihydroorotate dehydrogenase enzyme, and 6-azauracil, an orotidine 5′-phosphate decarboxylase enzyme inhibitor, effectively inhibit peste des petits ruminants virus replication in a JAK/STAT signaling-independent manner (228). Although a recent study determined dependency on IRF1 for Brequinar-mediated ISG induction, the detailed mechanism of this response remains elusive (229).

### Retinoic acid (RA)-mediated direct ISG induction

RA, a transcriptionally active metabolite of vitamin A, functions through heterodimers of RA receptors (RAR) and retinoid X receptors (RXR) that can bind directly to specific regions in the promoters called RA response elements (RAREs) of RA-inducible genes (230). RA has been detailed by numerous studies to show anti-viral properties against a wide range of pathogens (231–233). Interestingly, RA has been reported to induce ISGs at basal levels in Huh7 cells that are a poor producer of IFNs (234), suggesting IFN-independent regulation of ISG transcription by RA, possibly via RARE regions in the regulatory upstream elements of ISGs (231).

## FUNCTIONAL IMPORTANCE OF IFN-INDEPENDENT ISG EXPRESSION

The conventional paradigm of IFN-mediated antiviral response has few limitations. Firstly, viral pathogens have evolved a plethora of strategies to antagonize IFN induction and function (235, 236). Secondly, the elaborate signaling process allows the viruses enough time to replicate before an antiviral state is established. It thus makes early, IFN-independent pathways essential for rapid antiviral gene production. Non-canonical ISG induction enhances the host's antiviral defense by enabling rapid, tailored responses to viral threats, especially in vulnerable cells like stem cells and neurons (74, 237). For instance, basal ISG expression restricts numerous viral infections in human embryos (238), and HCMV infection (239), or HIV infections in hematopoietic progenitor cells (240). The intrinsic ISGs in glioblastoma cells (241), astrocytes (242), and neurons (169, 237) employ distinct mechanisms to inhibit viral replication protecting nervous tissue. This IFN-independent response in neurons is crucial due to the irreplaceability of mature CNS neurons.

In many cases, IFN-independent ISGs are sufficient to control infection when exposed to a limited number of viral particles without needing IFN production (243). For example, in epithelial and fibroblast cells low levels of viral infection can be prevented by IRF3-induced ISGs even before virus replication and without IFN or proinflammatory cytokine secretion, thus bypassing the inflammatory effects associated with IFN signaling (243). Only upon exceeding a certain threshold of viral exposure the IFNs and cytokine production is triggered, attracting immune cells and initiating a broader antiviral response (243).

Distinct sets of ISGs, expressed independently of IFNs, are tailored according to the cell- or tissue-type specific viral encounters. For instance, key ISGs confer resistance to IAV in respiratory epithelial cells (244), VSV in pancreatic adenocarcinoma cells (245), and HSV in epithelial cells (246). Similarly, IFN-independent ISG induction in the lungs of Syrian golden hamsters infected with SARS-CoV-2 shapes the lung's early response to viral replication prior to the stronger and prolonged activation by IFNs (247).

IFN-independent ISGs offer redundancy in the immune response, ensuring that the host has multiple layers of defense against viral infections, especially in cases where viral antagonists compromise or inhibit the canonical pathway. The diverse yet overlapping intrinsic signatures of ISGs in different cell types reflect the outcome of extensive host-pathogen interactions and selection pressures throughout evolutionary history. Additionally, IFN-independent ISGs, induced by paracrine signaling by the secreted factors from virus-infected dendritic cells (DCs), prime an antiviral state in the bystander DCs (248), thus preparing them to effectively combat an impending pathogen even before direct exposure.

## CONCLUSION AND FUTURE PERSPECTIVES

Non-canonical ISG induction provides a rapid, IFN-independent antiviral response, crucial for early defense against viral infections. This pathway operates quickly, often within specific cell types or in response to particular viruses, offering an immediate defense that buys time for the broader IFN-mediated defenses to take effect. The interplay between these two pathways ensures a layered immune response, with non-canonical induction acting as a basal safeguard that minimizes viral replication and spread. Their temporal dynamics are crucial but somewhat obscure. On one hand, the non-canonical pathways offer a rapid initial response, allowing cells to produce antiviral ISGs before the elaborate IFN-mediated canonical signaling is fully established. On the other hand, this pathway gets induced with delayed kinetics to support the ongoing or phasing out IFN response, like the U-ISGF3-mediated (47, 48) or IRF1-mediated antiviral program (57). This is also supported by biphasic regulation of pSTAT1, where other than an early RIG-I/MAVS/IFN-dependent phosphorylation of STAT1, a delayed IFN-independent STAT1 phosphorylation is detected in U5A cells (249). This gradual buildup and reinforcement of ISGs through sequential non-canonical and canonical induction ensures a robust antiviral state, tailored to the specific needs of different cell types and viral threats. It is unclear why some cells favor non-canonical over canonical ISG induction, and further research is needed to clarify their interplay. Also, detailed biochemical investigations are needed to elucidate the exact molecular pathways and spatiotemporal regulation of TFs involved in IFN-independent ISG induction.

Autophagy and cell death pathways, like apoptosis, necroptosis, and pyroptosis, serve as backup IFN-independent defenses against viral infections by eliminating infected cells and preventing virus spread (250–254). IRF1 regulates PANoptosis (255), and IRF3 participates in an IFN-independent antiviral response via apoptosis (256, 257), while STING can mediate autophagy and/or apoptosis in an IRF3/IFN-independent manner (258). However, how these TFs and ISGs modulate these pathways, particularly independent of IFNs, remains unclear. Viruses have also developed methods to counter these mechanisms. For instance, classical swine fever virus (CSFV) N^pro^ inhibits IRF3-mediated IFN-independent apoptosis (259), while HSV1's VP16 disrupts the IRF3-CBP transcription complex (260). Flaviviruses, like DENV (44, 123), tick-borne encephalitis virus (TBEV), and Langat virus (LGTV) (261), antagonize IFN-independent antiviral responses by various mechanisms, complementing their known ability to suppress IFN-α/β signaling. This ongoing battle illustrates the continuous evolutionary arms race between viruses and hosts.

ISGs exhibit direct and indirect antiviral effects against various pathogens, yet their precise mechanisms and coordination for effective antiviral defense, particularly those induced independently of IFN, remain underexplored. VSGs such as CMPK2 and C11orf83 act as potent antiviral proteins independently of IFN signaling (262, 263). While CMPK2 counters DENV infection in IFNAR-KO and STAT1-KO mice (262), C11orf83 exerts anti-VSV effects by activating the OAS3-RNaseL system without IFN production (263). In grass carp, two genes named *CiGig1* and *CiGig2* exhibit potent IFN-independent anti-grass carp hemorrhagic virus (GCHV) functions (264). These VSG-driven antiviral programs possibly augment IFN-dependent pathways or serve as backups to combat viral infections when IFN responses are undermined. Comprehensive molecular studies combining genetic, biochemical, and *in vivo* methods are essential to understanding ISGs' roles in viral infections. Research should explore ISGs' effects across infection stages, tissue interactions, and basal versus IFN-induced expression. Computational and experimental approaches can be very helpful in distinguishing VSGs from ISGs and elucidating their antiviral mechanisms (265). Aging has been linked to dysfunction of innate and adaptive immune responses, resulting in impaired pathogen defense and higher mortality and morbidity (266, 267). Research into how aging and autoimmune conditions impact IFN-independent ISG induction could help develop strategies to strengthen immune defenses in the elderly and those with autoimmune diseases. Genetic defects in IFN systems (268, 269) and autoantibodies against IFNs increase susceptibility to viral infections and adverse reactions to live-attenuated vaccines (270, 271). In these scenarios, the importance of IFN-independent ISG induction cannot be overstated. This underscores the scope for antiviral therapies targeting innate immunity components to activate IRF3, NF-kB, or other TFs and could potentially also induce IFN-independent immune responses, though further research is required to confirm this (272–274).

In a nutshell, both canonical and non-canonical pathways form a robust immune arsenal that cooperatively detects, neutralizes, and restricts the spread of viral infections, highlighting the intricate and dynamic nature of host-pathogen interactions. Dissection of non-canonical pathways could help discover novel therapeutic strategies for antiviral drug development, especially in cases of resistance to conventional therapies targeting the canonical pathway.
